# Potential of Mobile Technology to Relieve the Urgent Mental Health Needs in China: Web-Based Survey

**DOI:** 10.2196/16215

**Published:** 2020-07-07

**Authors:** Yuxi Tan, Ziwei Teng, Yan Qiu, Hui Tang, Hui Xiang, Jindong Chen

**Affiliations:** 1 Department of Psychiatry The Second Xiangya Hospital Central South University Changsha China; 2 National Clinical Research Center for Mental Disorders Changsha China; 3 Institute of Mental Health Central South University Changsha China

**Keywords:** mental health, mobile health, mobile phone, mobile app, needs

## Abstract

**Background:**

With the rapid development of information technology and mobile devices, an increasing number of mobile medical services and platforms have emerged. However, China’s current mental health situation necessitates further discussion and research on how to provide more patient-centered services in the face of many challenges and opportunities.

**Objective:**

This study aims to explore the attitudes and preferences of mental health service stakeholders regarding mobile mental health services and discuss the challenges and opportunities faced by mobile technology developers in China.

**Methods:**

A web-based survey was conducted by following the Checklist for Reporting Results of Internet E-Surveys (CHERRIES) checklist. A total of 586 valid questionnaires were collected. Respondents included 184 patients or their family members, 225 mental health professionals, and 177 people from the general population. Data analysis was completed using SPSS 24.0.

**Results:**

Among the various problems perceived regarding the current mental health medical environment, difficulty in finding appropriate psychologists and limited visit times ranked highest. Social media (n=380/586, 64.9%) was the most preferred platform among all participants, whereas professionals showed a higher preference for smartphone apps (n=169/225, 75.1%). Professional instruction, psychological consultation, and mental health education (ranked top 3) were the most commonly identified needs. Mental health professionals generally emphasized more on treatment-related mobile mental health service needs, especially medication reminders (χ^2^_2_=70.7; *P*<.001), symptom monitoring (χ^2^_2_=24.0; *P*<.001), and access to mental health resources (χ^2^_2_=38.6; *P*<.001). However, patients and their family members focused more on convenient web-based prescriptions (χ^2^_2_=7.7; *P*=.02), with the general population interested in web-based psychological consultation (χ^2^_2_=23.1; *P*<.001) and mental health knowledge (χ^2^_2_=9.1; *P*=.01). Almost half of the participants regarded mobile mental health services as highly acceptable or supported their use, but less than 30% of participants thought mobile mental health services might be very helpful. Concerns about mobile mental health mainly focused on information security. Service receivers also suspected the quality and professionalism of content, and mental health professionals were worried about time and energy consumption as well as medical safety.

**Conclusions:**

In terms of service flow, mobile services could be used to expand service time and improve efficiency before and after diagnosis. More individualized mobile mental health service content in more acceptable forms should be developed to meet the various needs of different mental health stakeholders. Multidisciplinary training and communication could be incorporated to facilitate the integration and cooperation of more well-rounded service teams. A standard medical record system and data format would better promote the development of future intelligent medical care. Issues such as ensuring service quality, solving safety risks, and better integrating mobile services with regular medical workflows also need to be addressed.

## Introduction

### Background

Of the 1.39 billion people in China, more than 16 million are affected by severe mental disorders [1]. The number of people who require professional intervention for any mental disorder is even higher, estimated at 230 million [1], that is, approximately 1 in 8 people in China have psychological problems and would likely benefit from psychological counseling. The mental health system in China is also facing many challenges, such as a low service utilization rate, unevaluated treatment effectiveness, limited access, and unbalanced allocation of resources [2].

There has been an explosive growth of app markets worldwide since 2015, with Android and iOS platforms releasing more than 165,000 health-related apps, approximately 7% of which target mental health issues [3]. The worldwide emergence of mobile apps in the field of mental health can be divided into 4 categories: evaluation, tracking or monitoring, treatment, and multitargets [4]. Many studies have reported the benefits of mobile mental health apps, such as improving symptoms and reducing recurrence [5]. However, there are still a number of problems related to service quality, data privacy, interoperability, etc. Stronger cooperation with medical institutions and more rigorously validated evidence regarding its effectiveness in addressing significant clinical symptoms are also necessary [6]. More research is needed to promote the formulation of standards and suggestions for better mobile mental health services, especially in the large untapped market of China.

In April 2018, the general office of the State Council of China promulgated the *Opinions on Promoting The Development of Internet + Medical Health,* and encouraged medical institutions to expand the space and content of medical services using the internet and other advanced information technologies. To improve the efficiency of medical services, this policy aims to build a smoother service process that covers prevention, diagnosis, and recovery while integrating web-based and offline medical services and to explore typical mobile health demonstrations based on artificial intelligence (AI) and intelligent health equipment that could realize real-time monitoring and evaluation of personal health conditions, such as chronic disease screening, early warning, and proactive intervention [7]. In December 2018, 10 ministries and committees of China jointly issued the *National Pilot Program for the Construction of a Psychosocial Service System*, which explicitly calls for psychological service networks and assistant service platforms for the whole society and encourages the use of information technologies, such as hotlines, networks, apps, and public accounts on social media platforms, to carry out science publicity and mental health assessment of common mental disorders, including depression and anxiety [8].

### Objectives

As mentioned, the use of advanced devices and technologies to provide more convenient mental and psychological services are not only urgent needs of the government and the public, but also inevitable trends of social development. However, whether the mobile services provided by enterprises, technology companies, or medical institutions can truly fulfill the needs of patients, family members, physicians, and the general population remains to be determined. Current studies on attitude or acceptance are mainly from countries with advanced electronic health (eHealth) experience, such as the United States, Australia, or Canada, which cannot represent the situation in China [9]. This study aimed to explore the needs and preferences for mobile mental health services of different stakeholders, including patients and family members, mental health professionals, and the general population, and to discuss challenges and opportunities for better mental health services based on China’s mental health situation.

## Methods

### Questionnaire Survey

An open web-based survey was designed following the Checklist for Reporting Results of Internet E-Surveys (CHERRIES) checklist [10], with an expert panel consisting of 4 clinical psychiatry professionals (HT, HX, YQ, and JC), and 2 academic mental health researchers (ZT and YT). The study was approved by the Institutional Review Board of the Second Xiangya Hospital, Central South University. The web-based survey was developed and tested by a well-known web-based survey tool called *Survey Stars*, which generated Quick Response (QR) codes and links to the questionnaire for distribution. Two versions of the questionnaire could be generated by the first appearing question asking what the participant’s role is: 1 questionnaire comprising 14 items targeted patients, family members, and the general population and 1 questionnaire comprising 12 items targeted mental health professionals. Each questionnaire started with a request for informed consent, a brief introduction of the researchers and aims, a notice of less than 5-min time consumption for participation, and information regarding privacy, which stated that no identifiable personal data would be collected and that all data would be under strict protection and accessible only to the researchers. The content of the questionnaire was designed based on existing mobile mental health services in China and the designers’ clinical experience, which consisted of 3 parts: (1) demographic characteristics, including gender, age, marital status, education, and economic status of the patients, family members, and the general population; mental illnesses that the patients, family members, and the general population focus on or are interested in; sex, age, and job title of mental health professionals; mobile device usage frequency and attitude toward current mental health services for all participants; (2) preference for web-based service platforms and service categories; and (3) perceived acceptance and helpfulness of, willingness to pay for or provide, and concerns about mobile mental health care (Multimedia Appendix 1). Single-choice, multiple-choice, and ranking questions were designed to fit the content. Ranking questions were automatically presented in a random order in the questionnaire to prevent bias.

### Participants and Procedure

With the aim of understanding the differences in needs and preferences between different stakeholders to help bridge discrepancies and increase acceptance for future mobile services, the sample was divided into 2 major groups: service providers and service demanders. Service providers consisted of mental health professionals, who are the most direct providers and are considered to have some authority and knowledge concerning what kinds of services are necessary and beneficial. Service demanders were divided into 2 types: (1) patients with mental illness from mental health institutes and their family members; and (2) individuals in the general population. Recent epidemiological data suggest that most mental disorders have become increasingly common across China in the past 30 years [1]. However, less than 7% of patients with mental disorders have pursued mental health care, which indicates that there are a multitude of unmet mental health needs among the general population and suggests the necessity to research their potential needs and preferences [11]. On the other hand, patients in contact with mental health care generally have severe symptoms and represent the largest proportion of mental health service demanders [11]. For the above reason, the attitudes of different service demanders were collected separately, with the assumption that there may be possible differences in needs and preferences between patients who have identified the need to see psychiatric doctors and the general population that might also have unidentified mental health needs.

Participants were recruited voluntarily, and the questionnaire was accessible to all visitors who opened the link or scanned the QR code. Incomplete responses and multiple entries (via Internet Protocol Address) were automatically checked by the *Survey Star* website to guarantee valid responses. A total of 586 completed questionnaires with the correct participation time were collected in July 2018 from the most popular social media platform in China, WeChat. The respondents included 184 patients and family members, 225 mental health professionals, and 177 people from the general population. Data from mental health professionals and the general population were mainly collected by sharing internet links on the authors’ WeChat. Data from patients and family members were mainly collected at a psychiatric outpatient department of a large general hospital through researchers’ face-to-face invitation to scan the QR code and fill out the questionnaire after participants demonstrated a willingness to participate and gave consent. A primary analysis demonstrated very similar results between patients and family members; thus, further analysis was conducted with these 2 groups of data merged as one.

### Statistical Analysis

Data analyses were performed using IBM SPSS version 24.0 (IBM Corp). Descriptive statistics were used to describe the study sample. Chi-square tests for single- and multiple-choice questions were applied to analyze whether the reported differences between the 3 groups were significant. Independent sample median tests were used for the preference ranking questions.

## Results

### Demographic Characteristics and Mobile Product Use

The basic information of all participants is presented in Tables 1 and 2. The general population tended to have a high proportion of women, young adults, higher education, good financial conditions, and expressed interest in common mental health issues, such as depression, anxiety, obsessive-compulsive disorder, and sleep disorders, whereas patients and family members were more concerned about severe illnesses, such as schizophrenia and mood disorders.

With regard to mobile device use, 83.2% (n=133/184) of patients and family members, 92.4% (n=208/225) of mental health professionals, and 91.5% (n=162/177) of respondents from the general population reported high or relatively frequent use. A significant difference of *P*<.001 between patients and family members and the 2 other groups was detected. The results are shown in Table 3.

**Table 1:** Demographic characteristics of the patients, family members, and the general population (N=361).

Characteristic		Patients and family members (n=184), n (%)	General population (n=177), n (%)	Chi-square (*df*)	*P* value
**Gender**				**7.5 (1)**	**.006**
	Male	83 (45.1)	55 (31.1)		
	Female	101 (54.9)	122 (68.9)		
**Age (years)**				**21.1 (6)**	**.001**
	<18	9 (4.9)	1 (0.6)		
	18-25	33 (17.9)	51 (28.8)		
	26-30	48 (26.1)	62 (35.0)		
	31-40	40 (21.7)	33 (18.6)		
	41-50	39 (21.2)	18 (10.2)		
	51-60	14 (7.6)	11 (6.2)		
	>60	1 (0.5)	1 (0.6)		
**Marital status**				**7.0 (3)**	**.06**
	Single	76 (41.3)	94 (53.1)		
	Married or living with partner	102 (55.4)	74 (41.8)		
	Divorced	5 (2.7)	7 (4.0)		
	Widowed	1 (0.5)	2 (1.1)		
**Education level**				**69.5 (5)**	**<.001**
	Elementary school and below	3 (1.6)	0 (0.0)		
	Junior high school	29 (15.8)	7 (4.0)		
	High school	43 (23.4)	9 (5.1)		
	Vocational school	40 (21.7)	26 (14.7)		
	Bachelor degree	54 (29.3)	85 (48.0)		
	Postgraduate and above	15 (8.2)	50 (28.2)		
**Economic status**				**29.4 (4)**	**<.001**
	Very poor	22 (12.0)	6 (3.4)		
	Fairly poor	30 (16.3)	7 (4.0)		
	Moderate	106 (57.6)	138 (78.0)		
	Fairly good	23 (12.5)	25 (14.1)		
	Very good	3 (1.6)	1 (0.6)		
**Disease focused/interested**				**N/A^a^**	**N/A**
	Schizophrenia	62 (33.7)	60 (33.9)		
	Bipolar disorder	32 (17.4)	37 (20.9)		
	Depressive disorder	29 (15.8)	128 (72.3)		
	Anxiety disorder	19 (10.3)	113 (63.8)		
	Obsessive-compulsive disorder	8 (4.3)	72 (40.7)		
	Phobia	1 (0.5)	33 (18.6)		
	Panic disorder	3 (1.6)	18 (10.2)		
	Eating disorder	0 (0.0)	10 (5.6)		
	Sleep disorder	13 (7.1)	86 (48.6)		
	Paranoid mental disorder	3 (1.6)	38 (21.5)		
	Schizoid affective disorder	3 (1.6)	16 (9.0)		
	Mental disorder caused by epilepsy	1 (0.5)	13 (7.3)		
	Mental retardation with mental disorders	1 (0.5)	15 (8.5)		
	Other	9 (4.9)	8 (4.5)		

^a^Not applicable.

**Table 2:** Demographic characteristics of mental health professionals (n=225).

Characteristic		Mental health professionals, n (%); (n=225)
**Gender**		
	Male	85 (37.8)
	Female	140 (62.2)
**Age (years)**		
	<18	0 (0.0)
	18-25	22 (9.8)
	26-30	53 (23.6)
	31-40	82 (36.4)
	41-50	44 (19.6)
	51-60	23 (10.2)
	>60	1 (0.4)
**Job title**		
	Chief physician/chief nurse	17 (7.6)
	Deputy chief physician/deputy chief nurse	25 (11.1)
	Attending physician/supervisor nurse	64 (28.4)
	Resident physician/nurse	57 (25.3)
	Assistant physician/nurse	22 (9.8)
	Medical related major student	28 (12.4)
	Other	12 (5.3)

**Table 3:** Frequency of mobile device use and perceived problems of mental health services in China (N=586).

Characteristic		Patients and family members (n=184), n (%)	Mental health professionals (n=225), n (%)	General population (n=177), n (%)	Chi-square (*df*)	*P* value
**Frequency of mobile device use**					**33.8 (8)**	**<.001**
	Very often	75 (40.8)	131 (58.2)	106 (59.9)		
	Fairly often	78 (42.4)	77 (34.2)	56 (31.6)		
	Moderate	24 (13.0)	13 (5.8)	15 (8.5)		
	Not often	7 (3.8)	1 (0.4)	0 (0.0)		
	Almost not use	0 (0.0)	3 (1.3)	0 (0.0)		
**Perceived difficulties of mental health services in China**					**142.2 (18)**	**<.001**
	Difficult to find a suitable psychological counselor or institution	49 (26.6)	105 (46.7)	115 (65.0)		
	Limited visit time with the doctor	80 (43.5)	99 (44.0)	84 (47.5)		
	Heavy economic burden	67 (36.4)	84 (37.3)	64 (36.2)		
	Heavy transportation burden for nonlocal patients	50 (27.2)	100 (44.4)	34 (19.2)		
	Difficult to make an appointment to a fixed doctor	29 (15.8)	71 (31.6)	42 (23.7)		
	Lack of simpler procedures for regular medicine purchase	40 (21.7)	77 (34.2)	23 (13.0)		
	Extremely long waiting time	47 (25.5)	39 (17.3)	32 (18.1)		
	Difficult to make appointments	46 (25.0)	35 (15.6)	28 (15.8)		
	Other	8 (4.4)	16 (7.1)	4 (2.3)		
**Preference for platforms**					**83.0 (12)**	**<.001**
	Social media (WeChat or QQ)	134 (72.8)	123 (54.7)	123 (69.5)		
	Smartphone apps	78 (42.4)	169 (75.1)	104 (58.8)		
	Text message	25 (13.6)	19 (8.4)	9 (5.1)		
	Phone call	35 (19.0)	45 (20.0)	16 (9.0)		
	Websites	21 (11.4)	36 (16.0)	30 (16.9)		
	Other	5 (2.7)	6 (2.7)	5 (2.8)		

### Perceived Problems With Mental Health Services

Regarding the current inconvenience of or the unmet needs for mental health services, the difficulty of finding a suitable psychologist (n=269/586, 45.9%) and the limited visit time with the doctor (n=263/586, 44.9%) were most frequently mentioned (χ^2^_18_=142.2; *P*<.001). Mental health professionals were more concerned about finding proper psychologists (n=105/225, 46.7%), the difficulty for patients from rural areas to visit the doctor (n=100/225, 44.4%), the limited visit time (n=99/225, 44.0%), the economic burden (n=84/225, 37.3%), the inconvenience of regular medicine purchase (n=77/225, 34.2%), and the difficulty of regularly seeing the same doctor (n=71/225, 31.6%). Patients and family members complained more about the limited visit time (n=80/184, 43.5%) and economic burden (n=67/184, 36.4%). Compared with the 2 other groups, they also expressed more dissatisfaction with the length of the waiting time (n=47/184, 25.5%) and the difficulty of making appointments (n=46/184, 25.0%). The general population emphasized psychological consultation (n=115/177, 65.0%), limited visit time in the clinic (n=84/177, 47.5%), and economic burden (n=64/177, 36.2%). The results are shown in Table 3.

### Preference for Web-Based Service Platforms

Among the frequently used platforms for web-based health services in China, there are social media such as WeChat and QQ, smartphone apps, text messages, phone calls, and websites. Social media (n=380/586, 64.8%) and apps (n=351/586, 59.9%) were most commonly chosen by the respondents. A total of 72.8% (n=134/184) of patients and family members reported a willingness to use social media and 42.4% (n=78/184) for apps, whereas 54.7% (n=123/225) of mental health professionals were more interested in the former and 75.1% (n=169/225) in the latter; the figures were 69.5% (n=123/177) and 58.8% (n=104/177), respectively, for the general population. The results are shown in Table 3.

There were significant differences in the preference toward mainstream social media among the 3 groups of participants (χ^2^_2_=17.0; *P*<.001). The higher interest in specific apps among professionals was statistically significant compared with other participants (χ^2^_2_=45.3; *P*<.001). With regard to more traditional means of communication, a higher proportion of patients and family members chose text messages compared with other groups (χ^2^_2_=8.1; *P*=.02), and the general population was less interested in using phone calls for mental health services (χ^2^_2_=10.5; *P*=.01). No significant difference in attitude toward websites was detected (χ^2^_2_=2.6; *P*=.28).

### Preference for Categories of Mobile Mental Health Services

**Figure 1:**
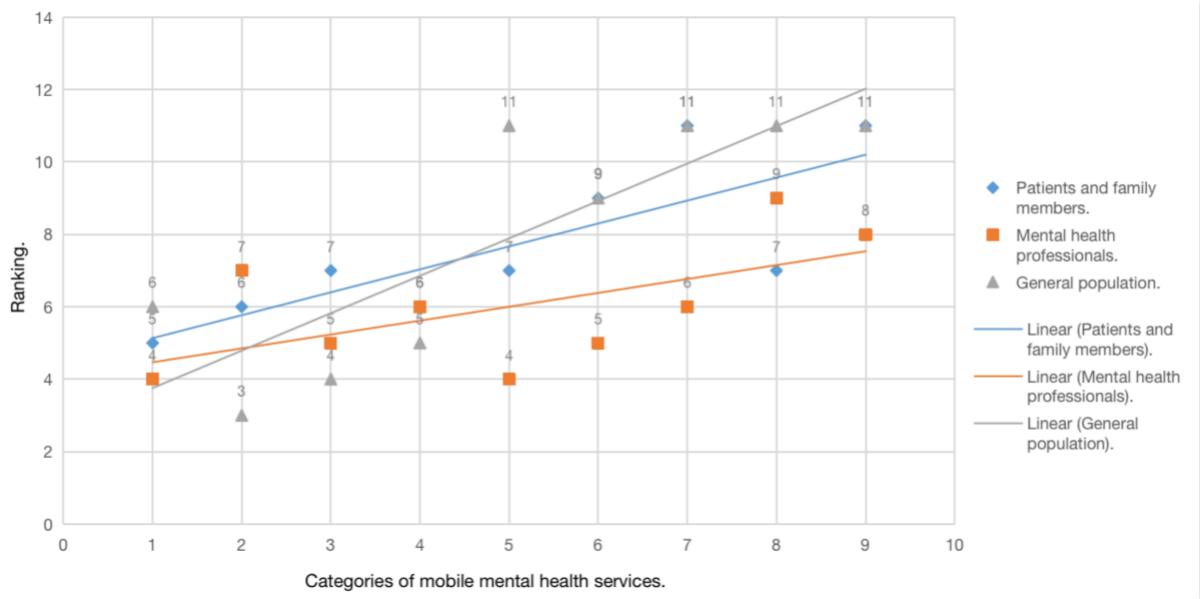
Median comparison of the ranking of different categories of mobile mental health services among the 3 groups. The X-axis: 1. Web-based professional instruction; 2. Web-based psychological consultation; 3. Mental health knowledge; 4. Guidance for healthy lifestyle; 5. Medication reminder and side effects monitoring; 6. Symptom monitoring; 7. Collection of mental health resources; 8. Web-based prescription; 9.Web-based peer support. The Y-axis represents the median ranking of each category of the 3 groups.

A multichoice ranking question was provided to determine whether there are differences among respondent groups regarding various categories of mobile mental health services. A total of 11 options were provided for ranking in the questionnaire including 9 categories of services, *others*, and not selected as the eleventh option. Table 4 presents the statistical differences of the priority given by the 3 groups for each category of mobile mental health service. Figure 1 provides a more visualized comparison of the median of each category’s ranking among the 3 groups. Numbers on the X axis correspond to the categories listed in Table 4. A smaller ranking number in the table or figure indicates a higher priority. Among all categories, the need for web-based professional instruction, web-based psychological consultation, mental health knowledge, and guidance for a healthy lifestyle ranked high, on average.

Except for guidance for a healthy lifestyle, all other items were significantly different among the 3 groups. Mental health professionals generally emphasized many items, especially medication reminders (χ^2^_2_=70.7; *P*<.001), symptom monitoring (χ^2^_2_=24.0; *P*<.001), mental health resources (χ^2^_2_=38.6; *P*<.001), and peer support (χ^2^_2_=19.0; *P*<.001). The general population attached much more importance to web-based psychological consultation (χ^2^_2_=23.1; *P*<.001) and mental health knowledge (χ^2^_2_=9.1; *P*=.01) but less importance to other demands, whereas patients and family members tended to focus more on convenient web-based prescription (χ^2^_2_=7.7; *P*=.02).

**Table 4:** Preferences for categories of mobile mental health services among the 3 groups.

Categories		Ranking^a^			Chi-square (*df*)	*P* value
		25th percentile	Median	75th percentile		
**Web-based professional instruction**					**6.6 (2)**	**.04**
	Patients and family members	2	5^b,c^	11		
	Mental health professionals	2	4^b^	9		
	General population	3	6^c^	11		
**Web-based psychological consultation**					**23.1 (2)**	**<.001**
	Patients and family members	2	6^c^	11		
	Mental health professionals	3	7^c^	11		
	General population	2	3^b^	11		
**Mental health knowledge**					**9.1 (2)**	**.01**
	Patients and family members	3	7^c^	11		
	Mental health professionals	2	5^b^	8		
	General population	2	4^b^	11		
**Guidance for healthy lifestyle**					**1.9 (2)**	**.38**
	Patients and family members	3	6	11		
	Mental health professionals	3	6	11		
	General population	2	5	11		
**Medication reminder and side effects monitoring**					**70.7 (2)**	**<.001**
	Patients and family members	3	7^c^	11		
	Mental health professionals	2	4^b^	7		
	General population	6	11^d^	11		
**Symptom monitoring**					**24.0 (2)**	**<.001**
	Patients and family members	3	9^c^	11		
	Mental health professionals	3	5^b^	9		
	General population	4	9^c^	11		
**Collection of mental health resources**					**38.6 (2)**	**<.001**
	Patients and family members	5	11^c^	11		
	Mental health professionals	3	6^b^	11		
	General population	5	11^c^	11		
**Web-based prescription**					**7.7 (2)**	**.02**
	Patients and family members	3	7^b^	11		
	Mental health professionals	4	9^b^	11		
	General population	5	11^c^	11		
**Web-based peer support**					**19.0 (2)**	**<.001**
	Patients and family members	5	11^c^	11		
	Mental health professionals	5	8^b^	11		
	General population	5	11^c^	11		

^a^The presence of b, c, and d of the median column indicates whether there are statistically significant differences in the median ranking among the 3 groups in each category. The same letter indicates no significant difference between groups; otherwise, there are significant differences. No b, c, or d means no significant difference was detected among all 3 groups.

^b^No significant differences between groups for these numbers.

^c^No significant differences between groups for these numbers.

^d^No significant differences between groups for these numbers.

### Acceptability and Concerns

Tables 5 and 6 show the acceptance and concerns regarding mobile mental health services. Nearly half of the participants regarded mobile mental health services as highly acceptable or recommended their use. However, in regard to perceived helpfulness, <30% of participants thought mobile mental health services might be very helpful, and almost half of them considered such services fairly helpful.

Most service receivers had an above moderate level of willingness to pay for mobile mental health services. The risk of leaked personal information and the professionalism of web-based services were frequently chosen as concerns with regard to these web-based services.

A total of 76.0% (n=171/225) of mental health professionals believed that mobile mental health services can be fairly or very beneficial to their clinical work. Given proper payment, most mental health professionals showed willingness to provide these services. However, almost half of the mental health professionals were concerned about the potential disadvantages of web-based services that were listed on the questionnaire, especially the danger of leaked personal information, insufficient time and energy investment, and medical safety.

**Table 5:** Acceptance of and concerns regarding web-based services of patients, family members, and the general population (n=361).

Characteristic		Patients and family members (n=184), n (%)	General population (n=177), n (%)
**Acceptance**			
	Very willing to accept	89 (48.4)	74 (41.8)
	Fairly willing to accept	50 (27.2)	53 (29.9)
	Indifferent	38 (20.7)	42 (23.7)
	Tend to not accept	7 (3.8)	7 (4.0)
	Not accept	0 (0.0)	1 (0.6)
**Perceived helpfulness**			
	Very helpful	50 (27.2)	46 (26.0)
	Fairly helpful	88 (47.8)	84 (47.5)
	Indifferent	39 (21.2)	41 (23.2)
	Tend to not be helpful	7 (3.8)	5 (2.8)
	Cannot be helpful	0 (0.0)	1 (0.6)
**Willingness to pay**			
	Very willing	33 (17.9)	29 (16.4)
	Fairly willing	64 (34.8)	60 (33.9)
	Indifferent	68 (37.0)	64 (36.2)
	Tend to not be willing	18 (9.8)	19 (10.7)
	Not willing	1 (0.5)	5 (2.8)
**Concerns**			
	Leakage of personal information	105 (57.1)	127 (71.8)
	Difficult to learn on mobile devices	60 (32.6)	77 (43.5)
	Difficult to keep recording and learning	66 (35.9)	84 (47.5)
	Not able to truly solve my problems	86 (46.7)	82 (46.3)
	Not professional or authoritative enough	71 (38.6)	98 (55.4)
	Other	6 (3.3)	7 (4.0)

**Table 6:** Acceptance of and concerns with web-based services among mental health professionals (n=225).

Characteristic		Mental health professionals (n=225), n (%)
**Support to use**		
	Very support	103 (45.8)
	Fairly support	69 (30.7)
	Indifferent	32 (14.2)
	Tend to not support	15 (6.7)
	Not support	6 (2.7)
**Helpfulness to patients and family members**		
	Very helpful	65 (28.9)
	Fairly helpful	104 (46.2)
	Neither helpful nor unhelpful	51 (22.7)
	Tend to not be helpful	3 (1.3)
	Cannot be helpful	2 (0.9)
**Helpfulness to work**		
	Very helpful	67 (29.8)
	Fairly helpful	104 (46.2)
	Neither helpful nor unhelpful	49 (21.8)
	Tend to not be helpful	3 (1.3)
	Cannot be helpful	2 (0.9)
**Willingness to provide with payment**		
	Very willing	80 (35.6)
	Fairly willing	62 (27.6)
	Indifferent	72 (32.0)
	Tend to not be willing	8 (3.6)
	Not willing	3 (1.3)
**Concerns**		
	Leakage of personal information	125 (55.6)
	Increased workload	105 (46.7)
	Not enough time and energy	145 (64.4)
	Medical safety is not guaranteed	132 (58.7)
	Unnecessary disturbance	107 (47.6)
	Other	4 (1.8)

## Discussion

What service providers prioritize may not be urgent from the perspective of service receivers. However, few studies have shed light on the needs concerning electronic-mental health (e-mental health) services from different stakeholders’ perspectives. Considering the current status of mental health in China and the fast-emerging eHealth trend worldwide, in-depth discussion is needed regarding the underlying contradiction between supply and demand in the current Chinese mental health services as well as the opportunities to solve these challenges and provide better patient-centered services through eHealth technologies.

### Acceptance and Preferences

In comparison with previous studies on acceptance and preferred mobile mental health services, the findings of this study shared some similarities, as expected. First, e-mental health services have been proven to be generally acceptable with a high willingness to use, even though they are not perceived to be as helpful as face-to-face services [12]. The results of this survey also indicate a high willingness to try such services alongside a relatively prudent expectation of e-mental health service helpfulness. Second, professional support has proved to be the most critical facilitator of user acceptability because both face-to-face and professionally guided web-based services are preferred over unguided self-help services [12]. The results of this study also show the high demand for services involving professional participation, such as professional medical instructions and psychological counseling.

This study adds to the body of literature in a few substantial ways. First, some studies have shown that low e-mental health awareness (e-awareness) and the digital divide of patients with severe mental illness might hinder the future use and perceived helpfulness of mobile services [13,14]. However, other studies have demonstrated that the internet is a significant resource for seekers of mental health knowledge and that the internet behavior of patients with psychosis is not very different from that of the general population [15]. The results of this survey supported the latter finding. The frequency of mobile device usage by patients and family members in this study was lower than that in the other 2 groups, but the respondents indicating above-average usage still accounted for 83.2%, and no significant difference in acceptance or willingness to use was found. There is still much space for developers to explore how to take full advantage of the cyber world and create more acceptable services for mental health seekers, regardless of the severity of their illness.

Second, previous studies have found that the vast majority of respondents preferred traditional face-to-face services to mobile mental health services, which put traditional medical services and mobile medical services on opposing sides [16]. However, the advantage of mobile medical services lies in their ability to assist and optimize traditional services rather than replace them. According to the reflection on current medical problems in China from this study, there are still many unmet needs, especially in terms of accessibility, service time, economic burden, and distance to institutions. This indicates that mobile mental health services have great potential to solve these medical needs that cannot be met at present, such as by offering more professional medical instruction and health education. Therefore, mobile health services need not be considered in opposition to traditional medical treatment but as advantageous to complement existing mental health services.

Third, to our knowledge, this is the first study on the preference for different types of mobile medical services and the first to compare among different stakeholders. Therefore, it is difficult to compare our findings with those arising from other countries’ situations, but the results of this study still provide valuable indications for the future development of mobile mental health services. This survey showed that mental health professionals generally place more emphasis on symptoms and treatment, such as monitoring and access provision, whereas service demanders are more focused on psychological counseling and convenient prescription. The emphasis of professionals on medical issues might be due to their tendency to diagnose and provide treatment plans based on symptoms, whereas psychological counseling services are not commonly integrated with current mental health institutions in China [2]. The higher requirements of patients and their family members for convenient prescription might be the result of the imperfect hierarchical diagnosis and treatment system in China [2], which leads to large provincial medical institutions often being crowded and inconvenient for rural patients to access. The obvious lower requirements of service demanders for monitoring and access provision could be due to the low adherence to medication, as shown in other research studies [17], but might also be due to the fact that service demanders believe they can monitor their symptoms and medications without additional mobile tools and instead give higher priority to items that they cannot accomplish alone, such as professional instruction and counseling. In short, this result reflects that the functions professionals think are important for mental health might not be considered of the same importance by patients and family members. This indicates that service developers should pay more attention to the different needs of different stakeholders and try to meet these differentiated needs in a more acceptable manner.

### Challenges

The urgent needs for professional instruction and psychological therapy seems to be intrinsic for patients and family members with mental illnesses, but many underlying problems in the current Chinese mental health environment provide no simple solution to these seemingly simple needs. eHealth services seem to be a promising solution, but there are some systemic challenges to be solved that are critical for their application and further development.

First, mental health human resources in China are limited. Due to the lack of social workers and family physicians and the imperfect hierarchical diagnosis and treatment system, patients are used to crowding into first-class hospitals for treatment. However, limited human resources and heavy work burdens make it difficult for physicians and nurses in hospitals to provide personalized and continuous disease management, which leads to the urgent need for more professional instruction, as shown in this study [2].

Telemedicine services seem to be a promising solution to help patients who lack convenient access to local services to obtain high-quality mental health services. The authors conducted a rough search on 2 App Store optimization websites, *Kuchuan* [18], and *Chandashi* [19] to obtain the number of recent mobile mental health apps available at the time of writing this paper. The results shown there are 222 of these apps in the App Store and 621 in the Android markets on *psychology (心理 in Chinese)*, and 212 and 121,560, respectively, on *psychological counseling (心理咨询 in Chinese)*. These results are in accordance with an industry report, where most of the high-ranked mental health service platforms provide web-based counseling [20]. These platforms continue to gather well-known psychiatrists and psychologists as much as possible to improve their popularity and professionalism [20]. There are also clinical trials demonstrating the effectiveness of mental health services provided on social media, such as WeChat [21].

However, there are several challenges that require further attention. On the one hand, the time consumption and workload disruption might hinder the sustainable willingness of more professionals to provide such services, as Granja and Janssen noted [22], which was also shown in this study as a major concern of professionals. On the other hand, the current lack of systematic training and standardized entry threshold for psychological counselors in China also leads to a lack of professional psychological counselors, which consequently makes psychological therapy an urgent need for the general population [2]. Additionally, there is no explicit regulation on the pricing of the psychological counseling industry in China. Current web-based psychological consultation fees are mostly hundreds of yuan, some even more than 2000 yuan (or approximately US $285) per hour, and none of these services are currently covered by medical insurance [23]. As a newly emerging industry, the medical and ethical safety of the web-based services provided by commercial companies is also awaiting further perfection of relevant laws and regulations. As a result, it is still doubtful whether a high-threshold unguaranteed psychological counseling can truly alleviate so many patients’ urgent needs.

Second, professional mental health publicity and education resources for the general population are insufficient. The various kinds of mental health information that flood the internet are not necessarily effective or safe. First, nonindividualized health education information is not as helpful as what personal physicians or counselors can provide for patients to solve their mental health problems [24]. Second, some negative unregulated psychology websites may completely misguide patients and even lead to death [25]. Service providers need to come up with more individualized and authorized health education, which can not only guarantee the effectiveness of information but also help information seekers avoid being inundated and misled by the massive amount of information.

Third, the inconformity of medical information impedes the development of medical big data. For a long time, the inconsistency of data standards and the irregularity of data formats have been important factors hindering the development of big data in China [26]. Medical systems in different regions and hospitals have different standards and formats in China, resulting in many data deficiencies and great difficulties in data cleaning. These information islands also hinder physicians from gathering information about the medical history of patients, which consequently affects treatment effectiveness. In addition, the lack of records of symptoms and medication use from daily life, such as mood fluctuations of patients with bipolar disorder and metabolic indicators of patients with schizophrenia, make it difficult to understand the course of patients’ disease in the short visit time with inconsistent information [27]. These are also urgent needs for professionals, as shown in this study. Although there have been many mobile apps aiming at offering ecological momentary assessment (EMA), which monitors adverse events and might help physicians understand the course of the disease, whether patients will accept such apps and how to guarantee adherence are problems that need to be resolved [27]. Nonetheless, if information from this monitoring system is summarized and reported to the health information systems in medical institutes in a unified and continuous manner, it might greatly facilitate diagnosis, treatment adherence, and rehabilitation.

### Opportunities

The survey showed that all groups generally have high acceptance for mobile mental health services, with different preferences. However, the application of mobile mental health in China still faces many challenges along with great opportunities to improve the current service flow, service content, and service quality. The following are some suggestions for the application of mobile mental health in China based on the worldwide trends of mobile health and China’s national conditions.

#### Optimize Service Flow for Different Stakeholders and Different Needs

To reduce the threshold and obstacles for the public to positively seek and accept mental health services, professional mobile platforms could be equipped to establish more coherent service flows. For the general population, there could be platforms providing mental health education, early symptom screening, self-help psychological intervention, and smooth referral services to professional psychological counseling or psychiatric institutions. For patients with severe disorders who need more systematic diagnosis and treatment, mobile devices can be used to provide pretreatment guidance before a formal visit by asking patients to fill in disease-related information in advance, which could not only provide more referential information for physicians in the very limited outpatient visit time but also expand the content of service and enhance the sense of being cared for. After the first visit with a clear diagnosis, web-based prescription and drug delivery services after video conferences could be used to change repeated brief follow-ups to high-quality web-based instruction, with all the information gathered from previous visits and daily EMA data on mobile platforms. Transforming the repetitive and tedious routine through web-based services might help reduce the travel time and economic burden of patients and relieve the crowded environment in large medical institutions.

It should be emphasized that to ensure the acceptance and perceived usability among professionals, all of these mobile services should be optimized in the current mental health service mode without disturbing the regular workflow or increasing the burden of staff [20]. In addition, mobile mental health services should be integrated into people’s daily life habits as much as possible through common social media such as WeChat or QQ, which are preferred platforms for service demanders. The preference of professionals for using specialized smartphone apps rather than social media might be due to privacy, energy, and time concerns, which calls for careful design of service platforms. For example, a special processing mechanism that allows service requests to be transferred to professionals only when the background robot or assistants are not capable of dealing with them can be established. With specialized methods for information receiving channels and processes, structured information gathering and convenient disease management could be ideally realized to meet both scientific and clinical research needs.

#### Provide Individualized Service Content in Acceptable Forms

##### Psychological Consultation

Psychological intervention is one of the services of most concern for the public, patients, and family members. Web-based psychological intervention services may be self-helped or professionally guided, synchronous, or asynchronous [28]. Recently, another promising method that combines psychological consultation with AI-supported chatbots has become popular, providing companionship, listening, interpersonal skills, cognitive correction, and behavioral activation, even though there is a long way before such chatbots become natural and humanized enough [29,30]. With limited accessible services in China, self-help services might be cost-efficient, but human-supported services may have higher acceptance and adherence [24]. One possible solution worth exploring might be to integrate the convenience of a chatbot with auxiliary human support from trained service assistants without creating more workload to the currently limited professional mental health human resources. In addition, there should be different forms of services for illnesses of different severities. For common mild psychological problems in the general population, priority could be given to easier access to self-help services, such as mindfulness meditation exercises, self-help cognitive behavioral therapy, and telepsychological counseling. What is important is that these platforms are regulated properly, and referral links to reliable mental health institutions should be provided. For patients with more severe problems, there should be more disease-specific or even individualized psychological therapy services that are provided by authorized organizations and closely linked with stepped mental health systems from the community to the hospital level. Technologies integrated with virtual reality (VR), augmented reality (AR), or serious games can also be used to provide more vivid therapy scenes, such as for patients with cognitive impairment or specific phobia.

##### Psychological Education

At present, there are many search engines on the internet that provide a variety of mental health knowledge, but most of them are delivered either as a professional reference or with unguaranteed quality, which might lead to inaccurate self-diagnosis [28]. Additionally, website information is usually provided passively by users searching for possible relevant content and does not target individuals, thus having limited potential to solve problems properly. Smartphone apps, emails, or text messages can be easily ignored, and may not be interesting enough to ensure adherence without further human interaction. More individualized and professional education methods should be emphasized. For the general population, educational knowledge related to a healthy lifestyle and mental health could be actively provided using public web-based spaces such as social media and official group chats or chatrooms that belong to schools, organizations, or institutions, to increase the dissemination of mental health knowledge and decrease the sensitivity to discussing such issues. Content combined with current news or interesting practical skills or provided in the form of short videos or games may be more acceptable. For patients with more health care requirements, mobile platforms provided by strictly selected mental health institutions can be used to provide systematic, individualized content for patients in different stages of disease.

##### Professional Instruction and Peer Support

The lack of mental health human resources and limited visit time at clinics in China leads to individualized professional instruction for disease-related questions being the most important requirement of patients and family members. A stepped health care system could be introduced to mental health institutions by integrating AI technology and human professionals. After learning and training, the AI robot could be used to complete the screening and identification work and automatically answer some simple professional questions, but as a guarantee, medical assistants could be equipped to solve more complex problems that the robot cannot address. Important decision-making problems, such as prescription or emergencies, including suicide or self-injury, can be referred to a physician. With the help of mobile technology and AI, the burden of physicians could be reduced, and more personalized services could be provided.

Peer support has been considered an important way of improving mental health that provides more perspectives and support for patients and family members. Mobile devices are widely used social tools and educational platforms for peer support in synchronous or asynchronous forms, but the activation of, commitment to, access to, and effectiveness of mobile peer support apps await further research [31,32]. The unfamiliarity of peer support and its benefit, deeply rooted stigma, and relatively limited authority might have caused the lower preference in this study, and more peer-based apps and risks need to be discussed.

##### Symptom Management and Service Information

Symptom monitoring and medication reminders provided by mobile tools were not as prioritized by patients and family members as they were by mental health professionals. Professionals might habitually pay more attention to symptoms and side effects and hope to have a more comprehensive understanding of the variation of medical indicators. However, patients might think these are redundant and even reminders of pain. A mutually beneficial approach is necessary. Motivational research has proven that external and intrinsic factors, such as the sense of accomplishment and control, increase of personal benefit, and formation of habits, could facilitate adherence to mobile services [33]. This reminds us that these monitoring functions could be delivered in more interesting, interactive, and rewarding forms, such as games, or integrated into the conversational context on a regular basis, rather than simply requiring patients to provide information and complete complex missions. A passive data collection function embedded in mobile phones or wearable devices could also be utilized to ensure service adherence and acceptance.

In the past 15 years, China’s mental health services have greatly improved with the conduction of a national program named *Central Government Support for the Local Management and Treatment of Serious Mental Illness Project* or the *686 Program* that was initiated in 2004 [2]. Many financial subsidy policies have also been put forward for patients with severe mental diseases, especially for impoverished patients, to obtain affordable, even free treatment and rehabilitation services [2]. However, the accessibility of this service information is the key factor that determines whether patients can truly obtain help. In this study, service providers prioritized this, but service demanders did not. This might be because service demanders believe that they have the ability to freely and conveniently access the internet and obtain all needed information. The internet helps patients gain relevant information quickly, but providing this information in more authoritative and integrated platforms might improve the efficiency of accessing high-quality medical services without being misled by advertisements or unrelated information in the broad cyber world.

#### Increase the Quantity of Mental Health Services

Considering the current divided training system between psychiatry and psychology in China [34], web-based continuing education could help provide more convenient multidisciplinary training in fields such as psychiatry, psychological therapy, social work, and vocational rehabilitation. Technological innovations, such as AR and VR, can also contribute to more vivid and realistic education scenarios and help increase learning efficiency. Multidisciplinary communication facilitated by mobile technologies could also be incorporated to facilitate the integration and cooperation of more well-rounded service teams. Another important aspect of quality improvement depends on the interconnection of various health data, especially medical records from different medical information systems. It is necessary to establish unified clinical and scientific data standards that are consistent with electronic medical records and other major health information systems to increase the efficiency of big data scientific research and promote the continuous development of intelligent medical services. In addition, mobile mental health services should be combined with medical records as much as possible to avoid information islands, help promote more coherent service processes, and increase adherence. With regard to the suspicions of service quality as reflected in this survey, it is vital to continuously research the acceptability, usability, and effectiveness of all fast-emerging types of exploration in this constantly evolving world of technology.

### Limitations

There are some limitations to this study. First, respondents representing mental health professionals and the general population were collected from the social media accounts of the researchers, and the respondents representing patients and family members are mainly collected from one mental health department of a provincial hospital. The similar background of the participants limits this survey in gathering perspectives from community-level medical institutes or patients with limited resources or literacy. Multicentered research is needed for a more well-rounded understanding. Second, this study is conducted with the prerequisite that all participants have smartphones and use WeChat and thus cannot address the needs of people who do not frequently use smartphones or WeChat. However, with the rapidly increasing number of cyber citizens and the large number of WeChat users in China, understanding the needs of this part of the public first is more feasible and promising. Third, it was unpractical to conduct an assessment on cognitive ability before consent was obtained for a questionnaire, and there is a possibility that some patients did not fully understand the questionnaire items. However, as the results of patients and family members are very similar, we tend to believe that there is only a minor possible influence.

### Conclusions

This study investigated the preferences, acceptance, and concerns regarding mobile mental health services of different mental health stakeholders and discussed the potential of mobile health services to relieve the urgent mental health needs of current China. Psychological therapy, professional instruction, and mental health education are most needed, which reflects the current problems of mental health services in China, such as the severely imbalanced supply and demand, doubtful and insufficient public education materials, incoherent mental health services flow, and the lack of standardization of medical information. However, challenges always accompany opportunities. With the high acceptability of mobile health services, mobile technologies have the potential to build a smoother mental health workflow, enrich urgently needed service categories, and improve the overall service quality. More practice and research are needed in the future to continuously maximize the advantages of technology while avoiding privacy risks and medical security issues.

## Appendix

Multimedia Appendix 1 Survey of preferences for mobile mental health services.

## Abbreviations

AI: artificial intelligence
AR: augmented reality
CHERRIES: Checklist for Reporting Results of Internet E-Surveys
eHealth: electronic health
e-mental health: electronic-mental health
e-awareness: e-mental health awareness
EMA: ecological momentary assessment
QR code: Quick Response code
VR: virtual reality
