# Supplementary material for: Potential of Mobile Technology to Relieve the Urgent Mental Health Needs in China: Web-Based Survey
Source: JMIR Mhealth Uhealth. 2020 Jul 7;8(7):e16215. doi: 10.2196/16215 (PMC7381064; doi:10.2196/16215)
Supplement: Multimedia Appendix 1 [file mhealth_v8i7e16215_app1.docx]

**Survey of Preferences on Mobile Mental Health Services**

**You are:**

1. Patients with mental illness
2. Family members/caregivers of patients with mental illness
3. Mental health professionals
4. General population

**Version One**

For Group 1 (patients with mental illness and family members/caregivers) and Group 3 (General population)

1. Gender
2. Age
3. Marital status
4. Education level
5. Economic status
6. Disease you focused on or interested in: [Single choice for group1. Multiple choice for group 3]

□ Schizophrenia

□ Bipolar disorder

□ Depressive disorder

□ Anxiety disorder

□ Obsessive-compulsive disorder

□ Phobia

□ Panic Disorder

□ Eating disorders

□ Sleep disorders

□ Paranoid mental disorder

□ Schizoid affective disorder

□ Psychiatric disorders caused by epilepsy

□ Mental retardation with mental disorders

□ Other:________

1. How often do you use mobile devices?

◌ Very often

◌ Fairly often

◌ Moderate

◌ Not often

◌ Almost not use

1. What do you think are the shortcomings of the current mental health services? [Multiple choice]

□ It is difficult to make appointments.

□ The waiting time for an appointment is too long.

□ The transportation burden is too heavy for non-local patients.

□ The visiting time with doctors is very limited.

□ The economic burden is too heavy.

□ It is difficult to make an appointment to a fixed doctor.

□ It is difficult to find a suitable psychological counselor or institution.

□ There lacks simpler procedures for regular medicine purchase.

□ Other:_________

1. What form/platform would you like to accept mobile mental health services? [Multiple choice]

□ Social media like WeChat or QQ, etc.

□ Specific smartphone application (App)

□ Text message

□ Phone call

□ Websites

□ Other:_________

1. What mental health services would you like to see on mobile devices (assuming they can be provided on mobile devices in the most ideal state)? [Ranking question, please fill in numbers in brackets in turn]

[] Useful mental health knowledge

[] Regular medication reminders and side effects monitoring

[] Regular symptom monitoring

[] Professional advice on developing healthy lifestyle

[] Online peer support

[] Online professional treatment and rehabilitation instruction

[] Online psychiatric prescription

[] Online psychological counseling

[] Collection of mental health resources

[] Other desired services:_________

1. To what extent are you willing to accept mental health services provided by mobile devices?

◌ Very willing to accept

◌ Fairly willing to accept

◌ Indifferent

◌ Tend to not accept

◌ Not accept

1. To what extent do you think the mental health services provided by mobile devices can help you?

◌ Can be very helpful.

◌ Can be of great help

◌ Indifferent

◌ Tend to not be helpful

◌ Cannot be helpful

1. Would you be willing to pay for mental health services provided by mobile devices?

◌ Very willing

◌ Willing to pay a certain fee

◌ Indifferent

◌ Tend to not be willing

◌ Very reluctant

1. What are your concerns about receiving mental health services on mobile devices? [Multiple choice]

□ Leakage of personal information

□ Difficult to learn on mobile devices

□ Difficult to keep recording and learning

□ Not able to truly solve my problems

□ Not professional or authoritative enough

□ Other:_________

**Thanks a lot for your participation!**

**Version Two**

For Mental health professionals (Group 2).

1. Gender
2. Age
3. Job title
4. How often do you use mobile devices?

◌ Very often

◌ Fairly often

◌ Moderate

◌ Not often

◌ Almost not use

1. What do you think are the shortcomings of the current mental health services? [Multiple choice]

□ It is difficult to make appointments.

□ The waiting time for an appointment is too long.

□ The transportation burden is too heavy for non-local patients.

□ The visiting time with doctors is very limited.

□ The economic burden is too heavy.

□ It is difficult to make an appointment to a fixed doctor.

□ It is difficult to find a suitable psychological counselor or institution.

□ There lacks simpler procedures for regular medicine purchase.

□ Other:_________

1. What form/platform would you like to provide mobile mental health services? [Multiple choice]

□ Social media like WeChat or QQ, etc.

□ Specific smartphone application (App)

□ Text message

□ Phone call

□ Websites

□ Other:_________

1. What mental health services would you like to see on mobile devices (assuming they can be provided on mobile devices in the most ideal state)? [Ranking question, please fill in numbers in brackets in turn]

[] Useful mental health knowledge

[] Regular medication reminders and side effects monitoring

[] Regular symptom monitoring

[] Professional advice on developing healthy lifestyle

[] Online peer support

[] Online professional treatment and rehabilitation instruction

[] Online psychiatric prescription

[] Online psychological counseling

[] Collection of mental health resources

[] Other desired services:_________

1. To what extent are you willing to support mental health services provided by mobile devices ?

◌ Very willing to support

◌ Fairly willing to support

◌ Indifferent

◌ Tend to not support

◌ Not support

1. To what extent do you think mental health services provided by mobile devices can help patients and their families/caregivers?

◌ It can be very helpful.

◌ Can be of great help

◌ Neither helpful nor unhelpful

◌ Tend to not be helpful

◌ Cannot be helpful

1. To what extent do you think mental health services provided by mobile devices can help with your professional work?

◌ It can be very helpful.

◌ Can be of great help

◌ Neither helpful nor unhelpful

◌ Tend to not be helpful

◌ Cannot be helpful

1. To what extent would you be willing to provide mental health services on mobile devices if you could charge a certain fee or remuneration?

◌ Very willing

◌ More willing

◌ Indifferent

◌ Tend to not be willing

◌ Not willing

1. What are your concerns about using mobile devices to provide mental health services? [Multiple choice]

□ Leakage of personal information

□ Increased workload

□ Do not have enough time or energy

□ Medical safety is not guaranteed

□ Unnecessarily disturbance

□ Other:_________

**Thanks a lot for your participation!**
